# Supplementary material for: Hard magnetic properties in nanoflake van der Waals Fe3GeTe2
Source: Nat Commun. 2018 Apr 19;9:1554. doi: 10.1038/s41467-018-04018-w (PMC5908800; doi:10.1038/s41467-018-04018-w)
Supplement: Supplementary file 1 — Supplementary Information [file 41467_2018_4018_MOESM1_ESM.pdf]

Hard magnetic properties in nanoflake van der Waals Fe_3GeTe_2

Tan *et al.*

Supplementary Note 1. The material characterization of Fe_3GeTe_2 crystal

Chemical composition

To analyze the chemical composition of synthesized Fe_3GeTe_2 (FGT) flakes, energy-dispersive X-ray spectroscopy (EDS) was carried out. As shown in Supplementary Figure 1a, we used bulk single crystalline FGT flakes after peeling off surface layers. EDS (Supplementary Figure 1b) revealed the FGT to be ~ 2.88:1:2.05 for Fe:Ge:Te.

Basic magnetic properties

A magnetic property measurement system (MPMS), which includes a superconducting quantum interference device (SQUID), was employed to characterize the magnetic properties of FGT. The volume of sample was 0.00012329 cm^3. Supplementary Figure 1d shows the temperature dependence of the inverse of magnetization. The data was collected in a magnetic field of 100 Oe, applied along the c-axis direction. We extracted the Curie temperature (T_c) from fitting according to the Curie-Weiss law in a magnetization graph, as shown in Supplementary Figure 1d.

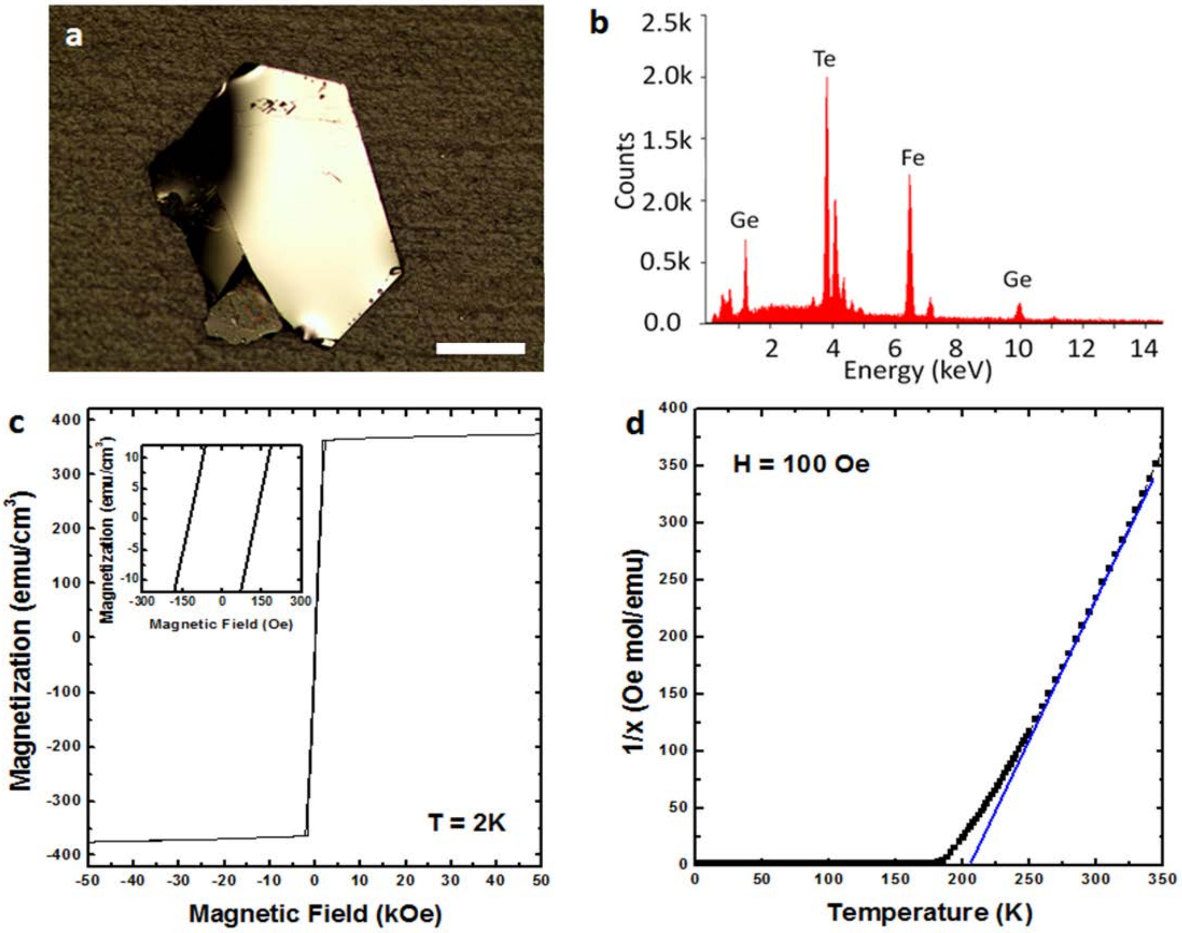

Supplementary Figure 1. Characterization of the FGT single crystal. (a) Optical image of a synthesized FGT single crystal. The white scale bar represents 1 mm. **(b)** EDS spectrum of synthesized FGT single crystal. The stoichiometric composition of three elements Fe, Ge, and Te is 48.59: 16.83: 34.58 for the synthesized crystal. **(c)** Field dependence of magnetization at 2 K. The calculated *M*s is 374.48 emu/cm^3. **(d)** Temperature dependence of inverse magnetization and fitted line for 100 Oe. The fitted line indicates the T_C = 205 K.

Supplementary Note 2. Detailed measurement data for other samples

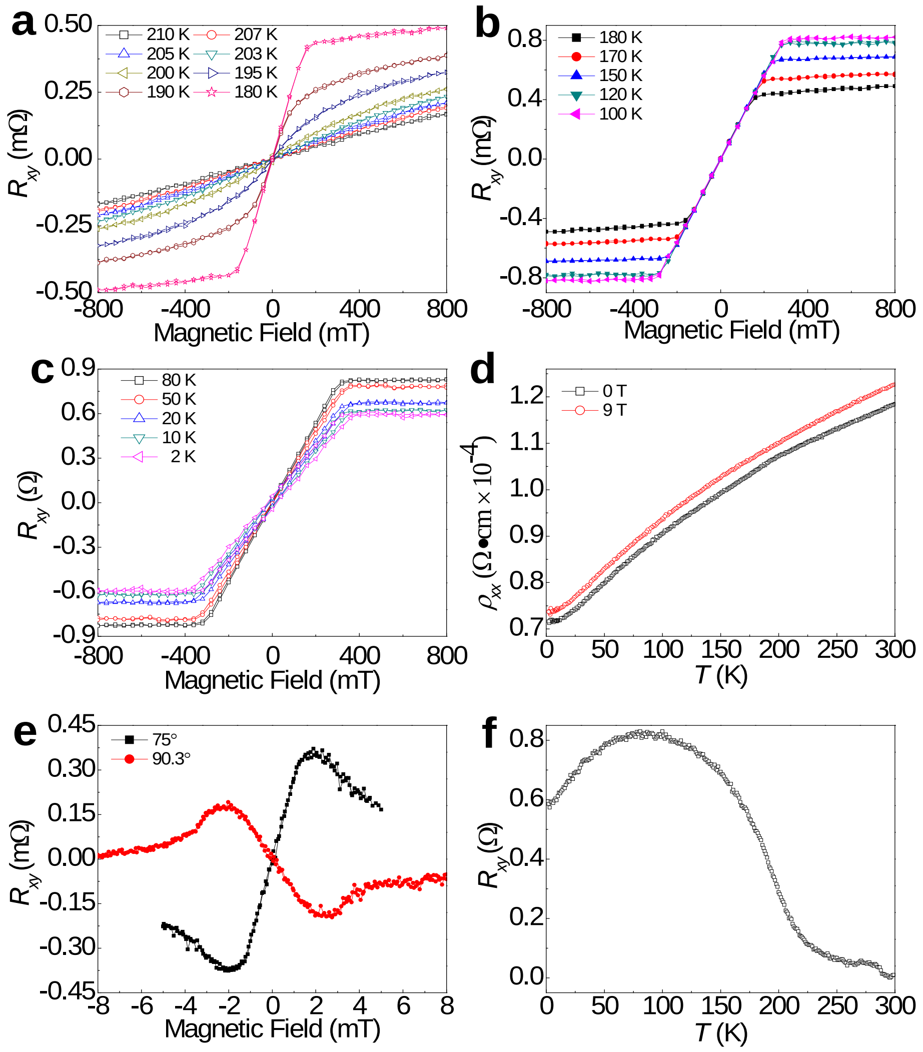

Supplementary Figure 2. R_{xy} and R_{xx} measurements for the bulk FGT single crystal. The contacts for the bulk device were made of Indium. **(a)** R_{xy} (B) curves show zero hysteresis in the temperature regime from 210 K to 180 K. The curve becomes linear at ~207 K. **(b)** R_{xy}(B) loops show no hysteresis from 100 K to 180 K. **(c)** R_{xy}(B) loops from 80 to 2 K. In this regime, the saturated magnetization decreases with decreasing temperature. At 2 K, the coercivity is ~21.6 mT. **(d)** Temperature dependence of ρ_{xx} at 0 T and 9 T. **(e)** Angular dependent Hall effect at 50 K with θ = 75° and 90.3°, respectively. When a high in-plane magnetic field (θ = 90°) is applied, the electron spins with perpendicular anisotropy will be forced parallel to the sample plane and then point to random direction when the magnetic field is swept back to zero, therefore the R_{xy} should be 0 in this condition. **(f)** R_{xy}(T) curve under 1 T field.

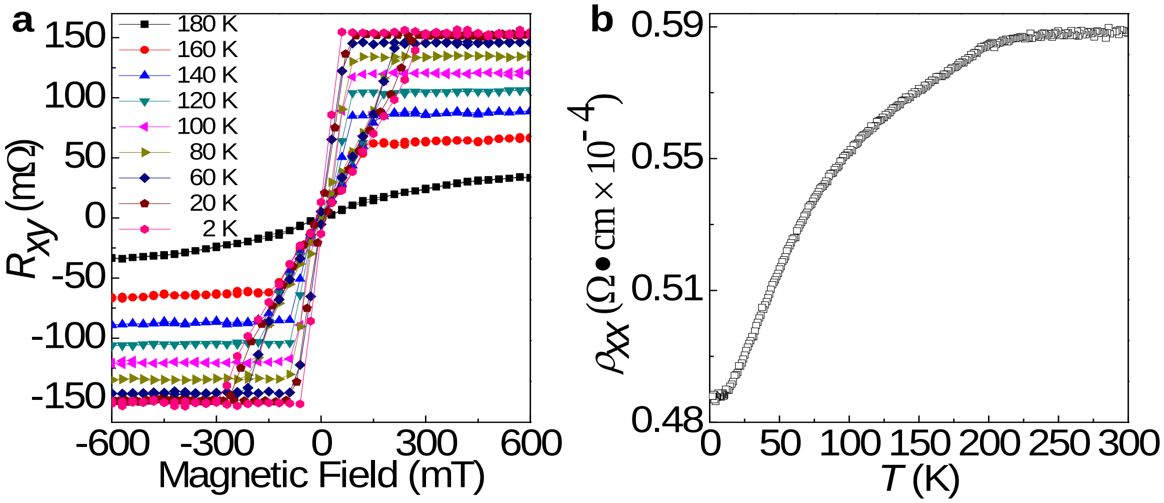

Supplementary Figure 3. $\boldsymbol{R_{xy}(B)}$ **and** $\boldsymbol{R_{xx}(T)}$ **curves for the FGT nanoflake with a thickness of 329 nm. (a)** $R_{xy}(B)$ curve at different temperatures. The hysteresis loops are not square. However, pronounced hysteresis loops with a non-zero coercive field are displayed. **(b)** $\rho_{xx}(T)$ curve at zero magnetic field, an obvious magnetic transition due to spin-flip scattering is shown at about 206 K, from which the Curie temperature is determined.

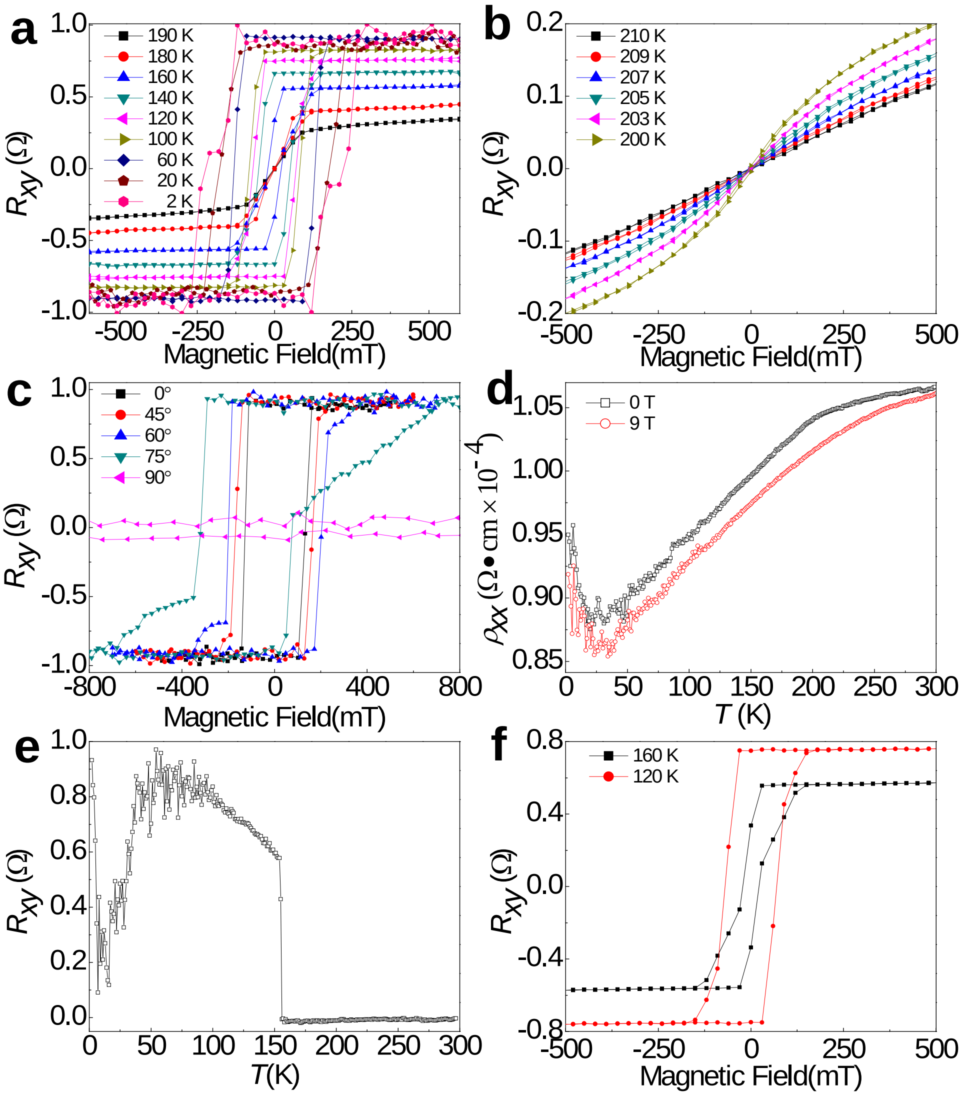

Supplementary Figure 4. R_{xy} and R_{xx} measurements for the nanoflake with a thickness of 191 nm. (a) $R_{xy}(B)$ loops from 2 K to 190 K. The coercivity increases with decreasing temperature. **(b)** $R_{xy}(B)$ curve from 200 K to 210 K. The curve becomes linear at ~209 K. All the curves show no hysteresis. **(c)** Angular dependent Hall effect at 50 K, the R_{xy} at 90° is nearly 0, which means the sample is nearly parallel to the magnetic field. At 75° an asymmetric loop is observed, which may indicate exchange coupling induced uni-directional magnetic anisotropy. **(d)** Temperature dependence of ρ_{xx} at 0 T and 9 T. Although the data is noisy at lower temperature, the spin-flip scattering is still clear. **(e)** Temperature dependence of the remanence. The sudden decrease of remanence indicates that the thermo agitation energy is higher than the perpendicular anisotropic energy, which can also be clearly seen from the evolution of the $R_{xy}(B)$ loop from 160 K to 210 K. **(f)** $R_{xy}(B)$ curve at 120 K and 160 K. Compared with the 120 K curve, the 160 K curve is indicative of two magnetic phases with increasing temperature.

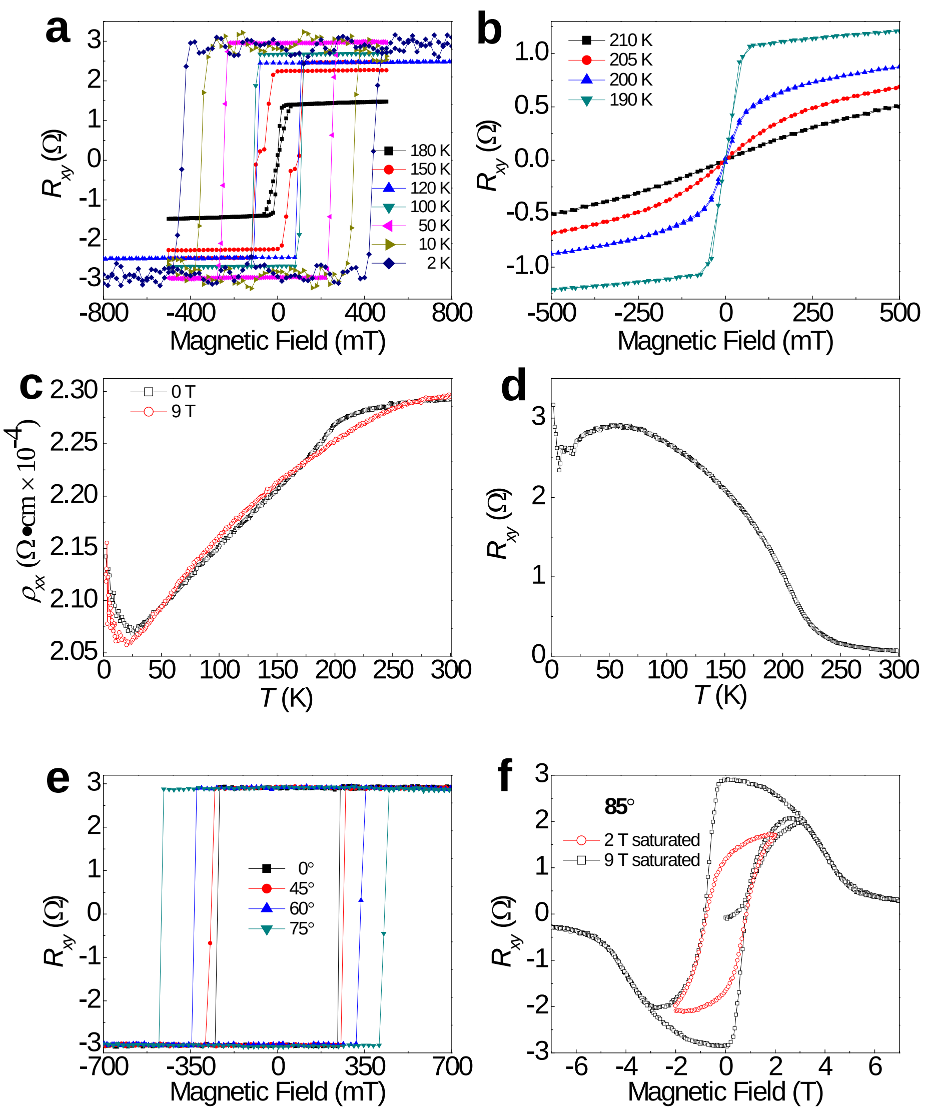

Supplementary Figure 5. R_{xy} and R_{xx} measurements for the nanoflake with a thickness of 82 nm. (a) $R_{xy}(B)$ loops from 2 K to 180 K. The 150 K and 180 K loops show behaviour indicative of two magnetic phases. **(b)** $R_{xy}(B)$ curve from 190 K to 210 K. The curve becomes linear at ~210 K. All curves do not show any hysteresis. **(c)** Temperature dependence of ρ_{xx} at 0 T and 9 T. Spin-flip scattering happens at ~200 K. **(d)** $R_{xy}(T)$ curve under 1 T applied field. **(e)** Angular dependent Hall effect at various angles (θ) at 50 K. **(f)** The angular dependent Hall effect at 85°. At first, the magnetic field was swept within 2 T, In this range, the curve did not show saturated behaviour. The $R_{xy}(B)$ shows saturated behaviour when the applied magnetic field exceeded 5 T, indicating a strong perpendicular anisotropy.

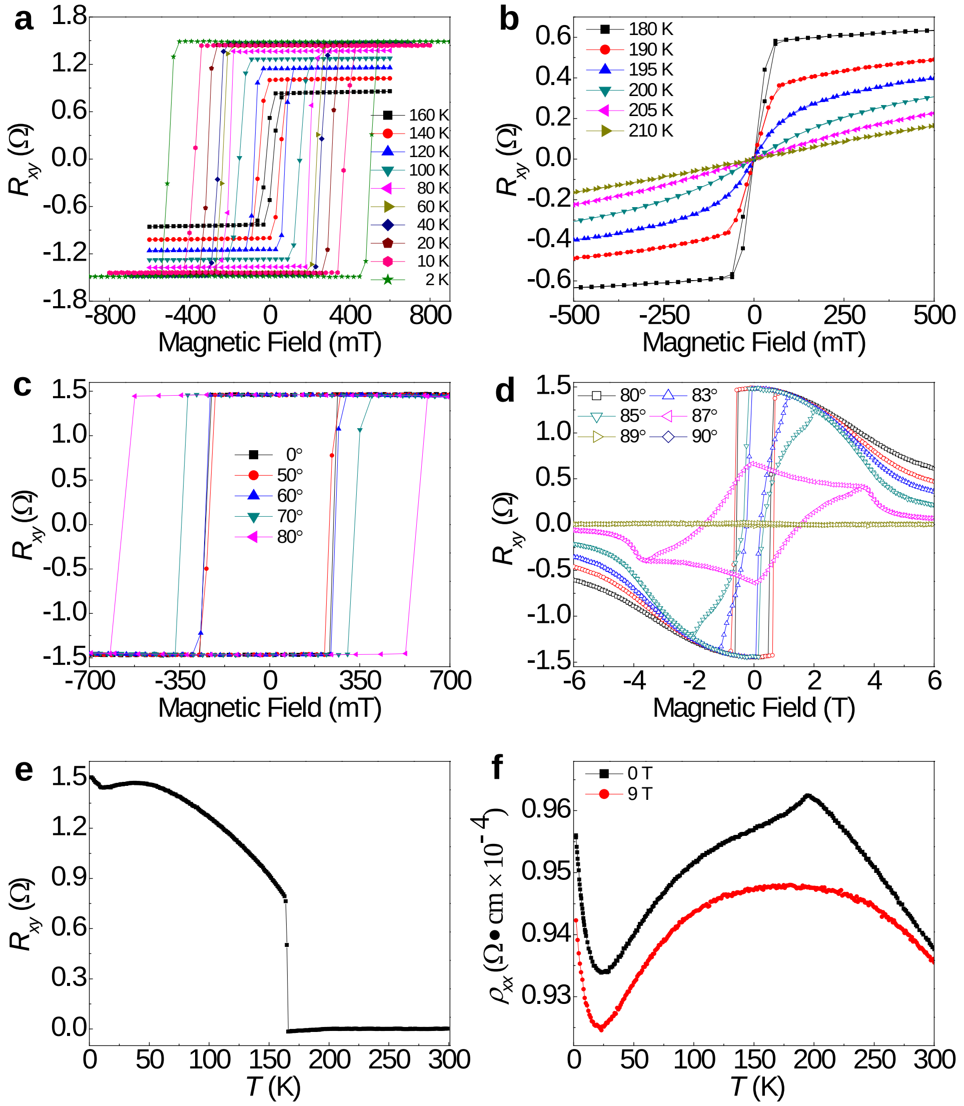

Supplementary Figure 6. R_{xy} and R_{xx} measurements for the nanoflake with a thickness of 49 nm. (a) $R_{xy}(B)$ loops from 2 K to 160 K. **(b)** $R_{xy}(B)$ loops from 180 K to 210 K. The curve becomes linear at 210 K. **(c-d)** Angular dependent Hall effect at 50 K. Loops between 0° and 50° are not shown, because they nearly overlap. Actually, the coercivity slightly decreases with increasing angle from 10° to 40°, which can be well fitted using the modified Stoner-Wohlfarth model, as described in Supplementary Note 4. **(e)** Temperature dependence of remanence. An obvious phase transition occurs at ~ 155 K. When T < ~20 K, the R_{xy} shows an abrupt rise. A phase transition may occur in this temperature regime. **(f)** The $\rho_{xx}(T)$ curve under zero magnetic field displays a peak at ~194 K, attributed to the spin-flip scattering near T_C. It can be supressed by a 9 T magnetic field as shown in the figure.

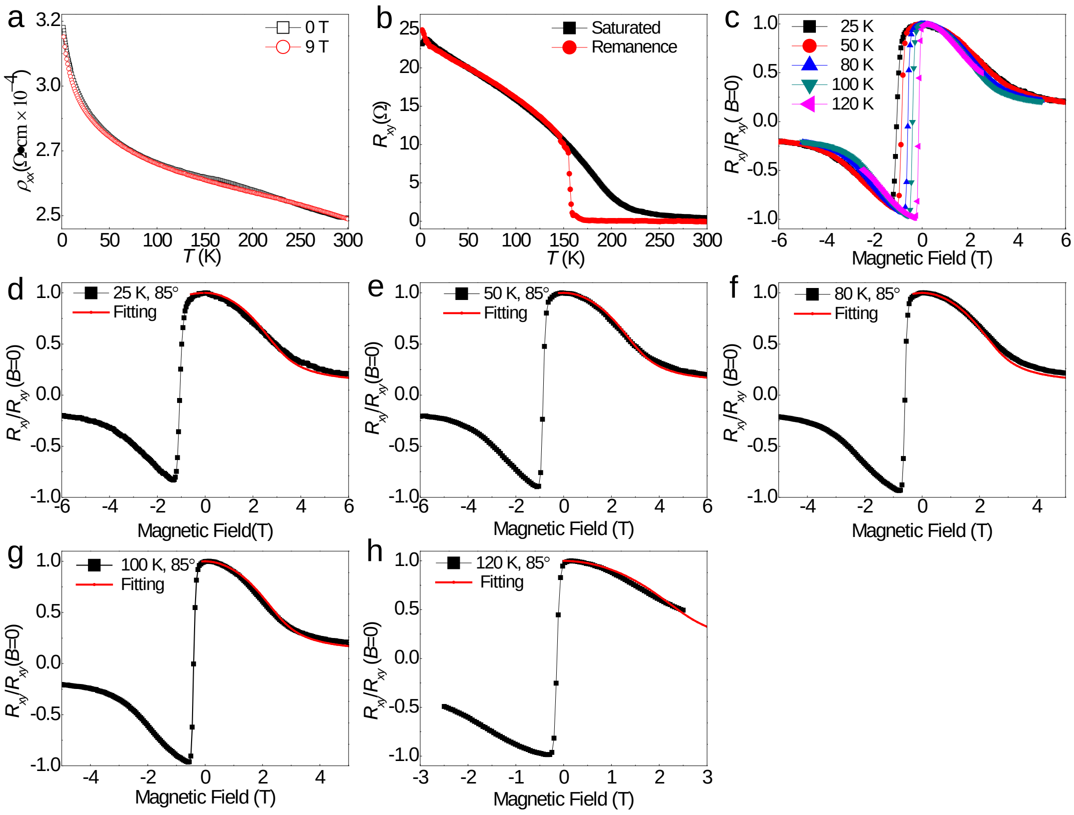

Supplementary Figure 7. Temperature dependent R_{xx} and R_{xy} loops from the nanoflake with a thickness of 10.4 nm. (a) Temperature dependence of the ρ_{xx} at 0 T and 9 T. Spin-flip scattering is evident near T_C. The enhanced resistance with decreasing temperature may originate from the disorder at the sample surface induced by exfoliation or oxidization. **(b)** Temperature dependence of remanence and R_{xy} curve with 1 T applied magnetic field. **(c-h)** Normalized angular dependent Hall resistance measured with the magnetic field swept from the positive saturated magnetic field to the negative saturated magnetic field at various temperatures. The red solid lines (d-h) are fitting curves based on the Stoner-Wohlfarth model.

Supplementary Note 3. Definition of the Curie temperature

The definition of Curie temperature is a vital part of magnetic material measurements. Generally, based on Curie-Weiss law, we can determine the Curie temperature of a sample through a linear fit to the temperature dependence of inverse magnetization above T_C. However, this method is only accurate when the critical exponent is 1 for the temperature-dependent susceptibility, which is unknown yet for FGT nanoflakes. Here we define the temperature at which the remanence R_{xy} goes to zero as T_C [the way similar to '*Nature* **546**, 265–269 (2017)'], which can give us more accurate T_C values.

In our experiments, the sample was firstly cooled down to 2 K under a magnetic field of 1 T (-1T). Then the magnetic field was slowly (5 Oe/s) decreased to 0 Oe at 2 K. Finally we scan temperature from 2 K to 300 K at 3 K/min and get the R_{xy} vs T curve with 1 T (-1 T) remanence. By telling the junction of remanence vs T curve of 1 T and -1 T (Supplementary Figure 8), where the remanence $R_{xy}(T)$ goes to zero, we can get the value of T_C.

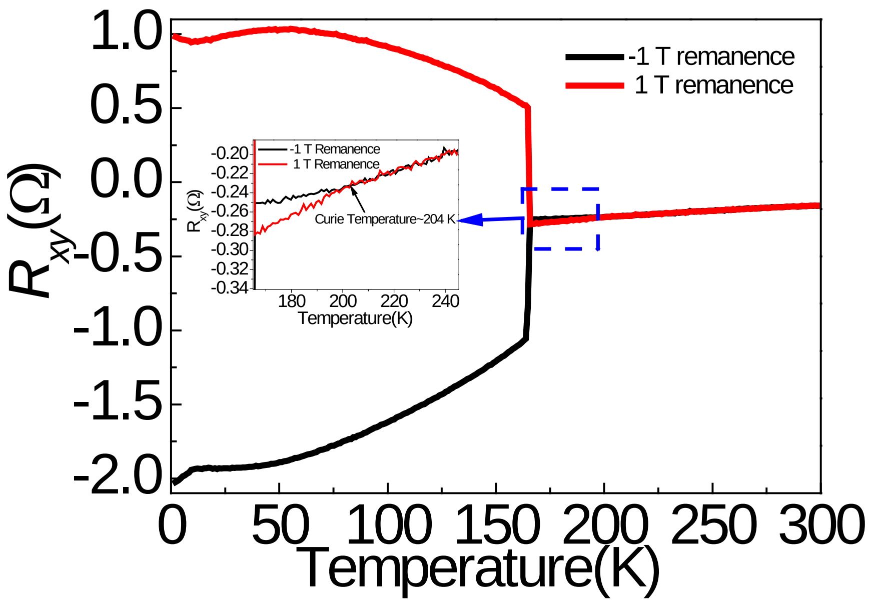

Supplementary Figure 8. Definition of the Curie temperature one FGT sample. The remanence curve of 1 T and -1 T saturation at 2 K meet at ~204 K, which we define as the Curie temperature. At this temperature, the remanence ($R_{xy}(T)$) becomes zero.

Supplementary Note 4. Anomalous Hall effect and the modified Stoner-Wohlfarth model

Anomalous Hall effect: basic formula and data process

In a ferromagnetic material, the relationship between the Hall resistance and applied magnetic field shows in Supplementary Equation 1.

$$R_{xy} = R_0 B_Z + R_S M_Z \tag{1},$$

Here, R_{xy} is the Hall resistance, which is composed of a normal Hall resistance (the first term in Supplementary Equation 1) and an anomalous Hall resistance (the second term in Supplementary Equation 1). B_Z and M_Z are the applied magnetic field and the sample magnetic moment perpendicular to the sample surface, respectively. The anomalous Hall effect is proportional to M_Z. As FGT is a metallic ferromagnetic material, the normal Hall resistance is very small compared with the anomalous Hall resistance in the magnetic field range of interest. Hence, the shape of the R_{xy} vs B loop is actually the same as that of the M_Z vs B loop when the applied magnetic field is perpendicular to the sample surface. The coercivity and $M_\mathrm{R}/M_\mathrm{S}$ ratio can be obtained from the $R_{xy}(B)$ curve.

Because of the non-symmetry in our nanoflake devices, the measured Hall resistance was mixed with the longitudinal magnetoresistance. We processed the data by using ($R_{xy\mathrm{A}}(+B)$ - $R_{xy\mathrm{B}}(-B)$) / 2 to eliminate the contribution from the longitudinal magnetoresistance, where $\mathrm{R_{xyA}}$ is the half loop sweeping from the positive field to the negative field, $R_{xy\mathrm{B}}$ is the half loop sweeping from the negative field to the positive field, and B is the applied magnetic field. We also measured the $R_{xy}(T)$ at the remanence point for all the samples. To measure the $R_{xy}(T)$ at the remanence point, the magnetic moment of samples was first saturated by a 1 T magnetic field and then the magnetic field was decreased to zero (the remanence point).

Finally, the temperature dependence of the R_{xy} at remanence was measured when the temperature was increased from 2 K to 300 K. In order to eliminate the non-symmetry effect of the device, we measured R_{xy} (remanence) with both 1 T and -1 T saturation. The real $R_{xy}(T)$ at remanence without R_{xx} mixing was calculated using ($R_{xy\mathrm{A}}(T)$ - $R_{xy\mathrm{B}}(T)$) / 2. Here $R_{xy\mathrm{A}}$ and $R_{xy\mathrm{B}}$ are the remenance with 1 T and -1 T saturation, respectively.

Demagnetization effect

The demagnetization effect is significant for FGT nanoflakes with perpendicular magnetic anisotropy. The following sentence is from '*Phys. Rev. B* **58**, 3223 (1998)', which shows the demagnetization factors for thin film with perpendicular anisotropy. "We approximate the thin film by a homogeneously magnetized ellipsoid of revolution of volume V whose radius $R_x = R_y = R$ is much larger than the 'film thickness' $2R_z$. The magnetostatic self-interaction energy is then given by $D\mu_0 M^2 V/2$, where $D \approx 1$ and $D \approx 0$ are the demagnetizing factors for in-plane and perpendicular magnetization orientations, respectively."

Based on the results above, we can obtain the effective field B_{eff} (magnitude and angle) from the applied magnetic field.

$$B_{eff} = \sqrt{(B\cos\theta)^2 + (B\sin\theta - M|\sin(\phi-\theta)|)^2}$$
$$\theta_{eff} = arctg\left(\frac{B\sin\theta - M|\sin(\phi-\theta)|}{B\cos\theta}\right) \quad (2)$$

The schematic diagram of the angular relationship of the applied field, magnetization, and perpendicular anisotropy is shown Supplementary Figure 9a.

Theoretical fitting by modified Stoner-Wohlfarth model

Three different energies, the magnetic anisotropic energy, the Zeeman energy due to the interaction between the applied magnetic field and magnetic moments, and the thermal

agitation energy determine the magnetic behavior of the FGT nanoflakes.

1. Fitting to the R_{xy} hysteresis loops at different temperatures

From the M_R/M_S ratio (~ 1), we know that all the spins in FGT nanoflakes align perpendicular to the sample surface at the remanence. Except the regime near the coercive field, an FGT nanoflake behaves like a single domain particle. Therefore, we can use the Stoner-Wohlfarth model to describe the magnetic behavior of a FGT nanoflake in the magnetic field regime away from the coercivity. As shown in Supplementary Figure 9a, the angles between the applied magnetic field and the direction of the perpendicular anisotropy and the magnetic moment are θ and ϕ, respectively. The direction of the magnetic field means the direction of the POSITIVE magnetic field.

Based on the Stoner-Wohlfarth model, the energy of a FGT nanoflake at temperature T can be written as

$$E(T) = K_A(T)V_S \sin^2(\phi - \theta_{eff}) - M_S(T)B_{eff}V_S \cos\phi \quad (3),$$

where K_A is the magnetic anisotropic energy, V_S is the volume of the sample, $M_S(T)$ is the magnetic moment of a unit volume FGT at temperature T, and B is the applied magnetic field. With an applied magnetic field B at known θ, we can easily calculate the ϕ value using the equation

$$\frac{\partial E(T)}{\partial \phi} = 2K_A(T)V_S \sin(\phi - \theta_{eff})\cos(\phi - \theta_{eff}) + M_S(T)B_{eff}V_S \sin\phi = 0 \quad (4),$$

In anomalous Hall measurements, the R_{xy} is proportional to the value of M_Z (the magnetization perpendicular to the sample surface). Therefore

$$R_{xy}(T) \propto M_S(T)\cos(\phi - \theta_{eff}) \quad (5).$$

By fitting the measured $R_{xy}(T)$ loops at temperature T based on Supplementary Equation 4, and Supplementary Equation 5, the magnetic anisotropic energy $K_A(T)$ can be obtained. The

most accurate $K_A(T)$ value can be fitted based on the loops with $\theta = 85^o$ as shown in Fig. 4c and Supplementary Figure 7(d-h). After the $K_A(T)$ value is obtained, the angular dependent coercivity can then be fitted based on a modified Stoner-Wohlfarth model at temperature *T*.

2. Fitting to the angular dependence of coercivity based on a modified Stoner-Wohlfarth model

When the magnetic field is swept to a negative field, the *B* field in Supplementary Equation 3 and Supplementary Equation 4 is negative.

To fit the angular dependence of coercivity, we make **two assumptions**,

1. Supplementary Equation 4 can have two kinds of solutions, the stable state (low energy) and the unstable state (high energy state), as shown in Supplementary Figure 9b. With an increasing magnetic field *B* (more negative *B* value), the energy difference ΔE between the meta-stable state 1 and the unstable state 2 decreases. At a certain *B* field, the thermal agitation energy is large enough to overcome the ΔE in a standard experimental time and the magnetic moment will flip to a stable state in the opposite direction. As the FGT nanoflake shows a nearly square-shaped R_{xy} loop (magnetic loop), we can assume that this *B* field is the coercive field.
2. When the first domain flips to the opposite direction under an applied magnetic field, other un-flipped magnetic moments will generate an effective field on the magnetic moment in the first flipped domain.

An important issue for the assumption 1 is to determine the ratio $\Delta E/k_B T$ to realize the experimentally observable flipping of domains. Neel and Brown proposed that the relaxation time τ for the system to reach thermodynamic equilibrium from the saturated state can be written as

$$\tau^{-1} = f_0 Exp(-\Delta E / k_B T) \quad (6),$$

where f_0 is a slowly variable frequency factor of the order 10^{-9} sec^{-1}. Assuming $\tau = 100$ Sec, Supplementary Equation 6 yields the condition

$$\Delta E = 25k_B T \quad (7),$$

Based on Supplementary Equation 3, 4 and 7, we can write the equations to fit the angular dependence of coercivity at temperature T.

$$2K_A(T)\sin(\phi_2 - \theta_{eff})\cos(\phi_2 - \theta_{eff}) + M_S(T)B_{eff}\sin\phi_2 = 0 \quad (8a),$$

$$2K_A(T)\sin(\phi_1 - \theta_{eff})\cos(\phi_1 - \theta_{eff}) + M_S(T)B_{eff}\sin\phi_1 = 0 \quad (8b),$$

$$[K_A(T)V(T)\sin^2(\phi_2 - \theta_{eff}) - a(T)V(T)M_S(T)B_{eff}\cos\phi_2] - [K_A(T)V(T)\sin^2(\phi_1 - \theta_{eff}) - a(T)V(T)M_S(T)B_{eff}\cos\phi_1] = 25k_B T \quad (8c),$$

where $V(T)$ is the volume of the first flipped domain at T, $a(T)$ is the parameter describing the effective field due to the coupling between the first flipped domain and the un-flipped magnetic moments at temperature T, which affects the Zeeman energy. The value of $a(T)$ should be between 0 and 1. ϕ_1 and ϕ_2 are the angles between the applied magnetic field and the magnetic moment for the meta-stable state 1 and unstable state 2, respectively, which are NOT fitting parameters. They were calculated using the Supplementary Equation 8a and 8b. The domain dynamics in the system is actually very complex. We should not think that the first flipping domain flips to ϕ_2, while the magnetic moments of other parts of the FGT flake still points to the ϕ_1 direction. As the magnetic loop of FGT is square-shaped, all the magnetic moments flip and overcome the barrier (ϕ_2) in a very narrow range of magnetic field. In this situation, using the calculated ϕ_1 and ϕ_2 based on Supplementary Equation 4 is a pretty good approximation.

$V(T)$ and $a(T)$ are the only two fitting parameters of the three equations in Supplementary Equation 8, by which the experimental angular dependence of coercivity at various

temperatures can be well fitted , as shown in Fig. 4d. The fitted $V(T)$ and $a(T)$ are also very reasonable. All the $a(T)$ values are between 0 and 1, which means the effective field decreases the effect of the Zeeman energy (a < 1), while it cannot totally eliminate the effect of Zeeman energy (a > 0). The temperature dependence of $a(T)$ shown in Supplementary Figure 9d can be explained as below. With increasing temperature, the coercive field decreases and therefore the $\phi - \theta$ decreases. The ratio between the energy due to the coupling of the flipped domain and the un-flipped magnetic moment to the Zeeman energy increases. Hence, a (T) decreases with an increasing temperature.

It should be emphasized that the fitted $V(T)$ and $a(T)$ values are the only possible values to fit the experimental data. Small deviations from the fitting results generate a large difference between the fitting curve and the experimental data (Supplementary Figure 9c), which clearly demonstrates that our model provides a reasonable description of the magnetic phenomena in FGT nanoflakes.

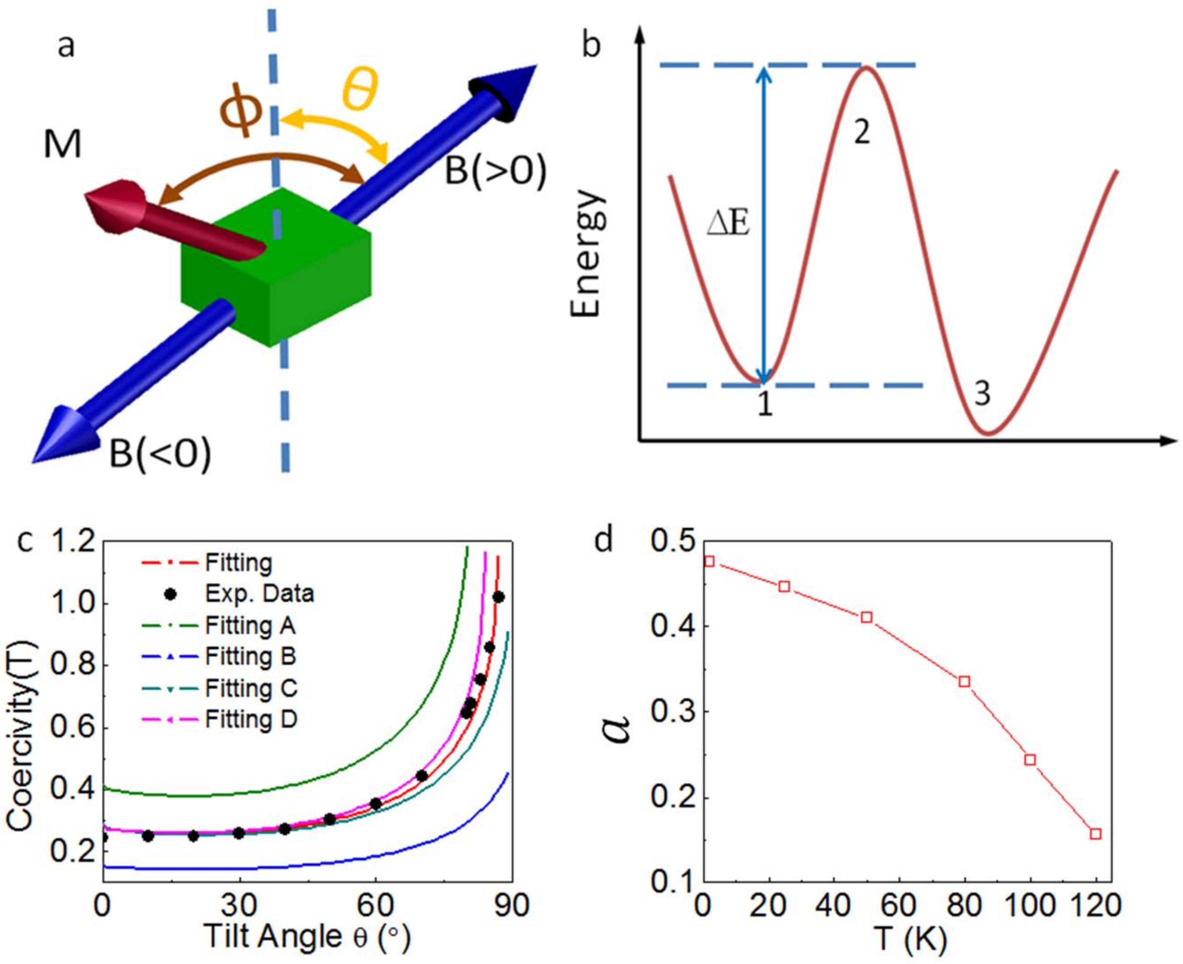

Supplementary Figure 9. (a) Schematic of the Stoner-Wohlfarth model. **(b)** Schematic diagram of a magnetic system change from stable to unstable state. **(c)** Experimental and fitting curves with different values of V and a (K_A=4.53 × 10^6 erg/cc). Fitting: V = 481 nm^3, a = 0.407. Fitting A: V = 550 nm^3, a = 0.407. Fitting B: V = 430 nm^3, a = 0.407. Fitting C: V = 481 nm^3, a = 0.46. Fitting D: V = 481 nm^3, a = 0.36. **(d)** The fitted parameter describing the effective field generated by the interaction between the first flipped domain and the un-flipped magnetic moments.

Supplementary Note 5. Mean field and spin wave fittings

To fit the temperature dependent remanence in Fig. 3c, we tried the mean field theory (the Brillouin function) and the spin wave theory.

The Curie-Weiss mean field theory

Due to the interaction between magnetic moments, an internal field can be written as

$$B_i = n_w M_{sp} \quad (9)$$

where n_w is a parameter describing the strength of the internal field and M_{sp} is the spontaneous magnetization.

$$M_{sp} = M_0 \Im_J(\mathrm{x}) \quad (10)$$

$$x = \frac{M_0 B_i}{k_B T \cdot N} \quad (11)$$

Where M_0 is the magnetic moment at zero K, $\Im$ is Brillouin function, k_B is Boltzman constant, N the total number of unit magnetic moments. From the above equations, we easily obtain

$$\frac{M_{sp}}{M_0} = \Im_J(x) \quad (12)$$

$$\frac{M_{sp}}{M_0} = \frac{x \cdot N \cdot k_B T)}{n_w M_0^2} \quad (13)$$

Now we calculate the value of T_C

$$n_w M_{sp} \cdot M_0 \approx k_B T_C \quad (14)$$

Therefore, we obtain

$$n_w M_0 \cdot \Im(x) \cdot M_0 = k_B T_C \quad (15)$$

We obtain $n_w M_0^2 \frac{(J+1)}{3J} \approx k_B T_C$ (16)

Combine Supplementary Equations 11, 12 and 16, we can easily calculate the M_{sp} vs (T/T_C) curves for different J values.

Spin wave model

The temperature dependence of magnetic moments of a three dimensional spin wave is $M \propto a + b(\frac{k_B T}{2J_s})^{3/2}$, which has been discussed in many text books.

Supplementary Note 6. The effect of the surface amorphous oxide layer

Though we tried to minimize the exposure of samples to ambient conditions, but a significant oxide layer could still form quickly on the top of the nanoflakes, which has been confirmed by cross-sectional electron microscopy images shown in Supplementary Figure 10a.

We choose the same time of ambient exposure as that in our device fabrication and transport measurements (~7 mins). The image shows that there is indeed an amorphous oxide layer of ~1.2 nm thick on the sample surface.

To check whether FGT flakes still show hard magnetic phase with a near square-shaped loop, we fabricated ultraclean devices using our new vdW fabrication system in a glove box with both O_2 and H_2O < 0.1 ppm. The image of one of the devices is shown in Supplementary Figure 10c. To fabricate this device, we utilized the method in '*Nat. Phys.* **13**, 677–682 (2017)'. Firstly, 5 nm thick Pt contacts were fabricated on Si/SiOx substrate in ambient condition. In our glove box, an exfoliated FGT flake was then dry transferred onto the contacts to form very good ohm contacts. Thereafter, a large hBN flake was dry transferred to cover the FGT flake to prevent oxidations in measurements. Finally, the sample was covered by PMMA to prevent any possible oxidization. The R_{xy} vs H loop is shown in Supplementary Figure 10d. It is very clear that the ultra-clean FGT flake still shows hard magnetic property with square shaped loop. The R_{xy} vs T curve also shows the same property as the device described in the main text. Thus, the thin oxide layer on the sample surface does not affect the main conclusions (hard magnetic properties with a near square-shaped loop) of this paper. Moreover, we can see that FGT is a promising material whose magnetism can survive in ambient environment for a certain time.

From our experiments, we conclude that the effect of oxide layer includes:

1. The switch of magnetic moment in the square shape loop of FGT with oxide layer is not as sharp as that in ultra clean FGT flakes, which is due to the pinning effect of the oxide layer.

2. The coercivity of FGT slightly increases after the oxidization, which is also due to the domain wall pinning effect.

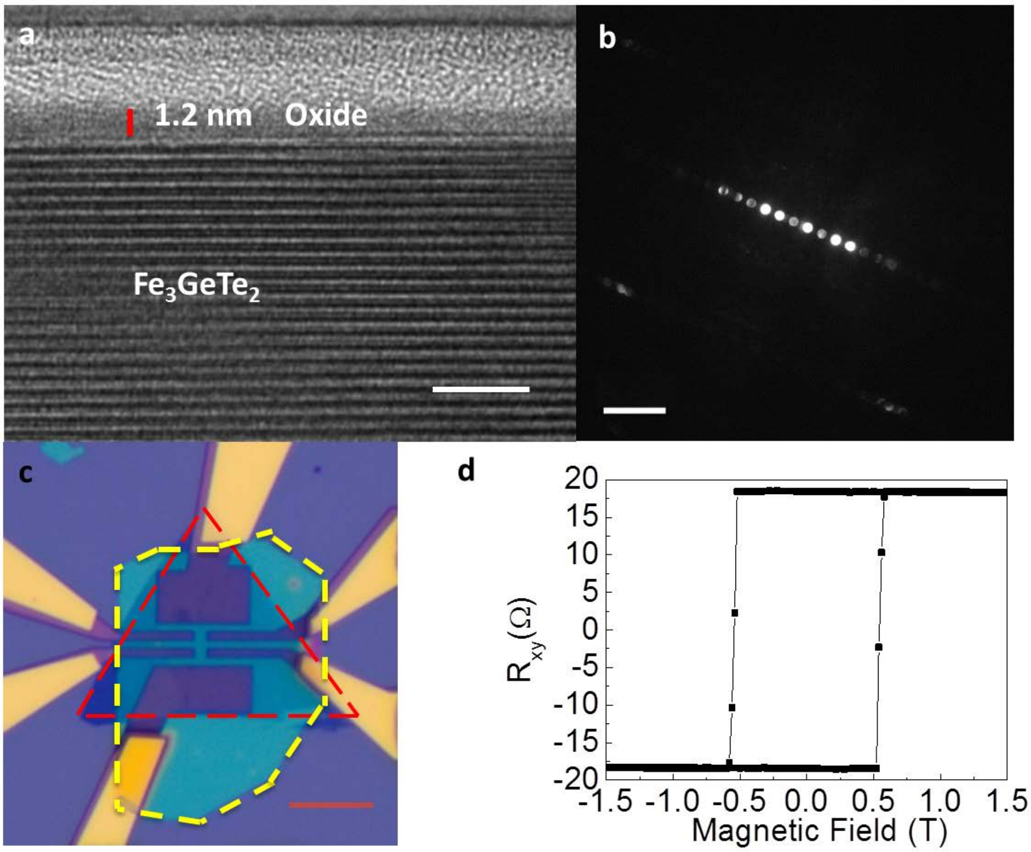

Supplementary Figure 10. (a) Cross-sectional TEM image of the FGT nanoflakes on substrate. The top layer is 5 nm Pt layer. The oxide layer is about 1.2 nm. The thickness of monolayer is about 0.8 nm. The scale bar represents 5 nm. **(b)** Diffraction pattern of the FGT nanoflake. The scale bar represents 5 nm^{-1} **(c)** A 5.8 nm FGT device covered by hBN, the bottom contact is 5 nm Pt. The red dashed line is FGT region, yellow dashed line is h-BN region. The red scale bar is 10 μm. **(d)** Anomalous Hall effect at 2 K for this device. Magnetic field is perpendicular to the sample surface.

Supplementary Note 7. The confirmation of ohmic contact

We fabricated an FGT device using the same recipe as all the other samples and confirmed our procedure of device fabrication produced good ohmic contacts.

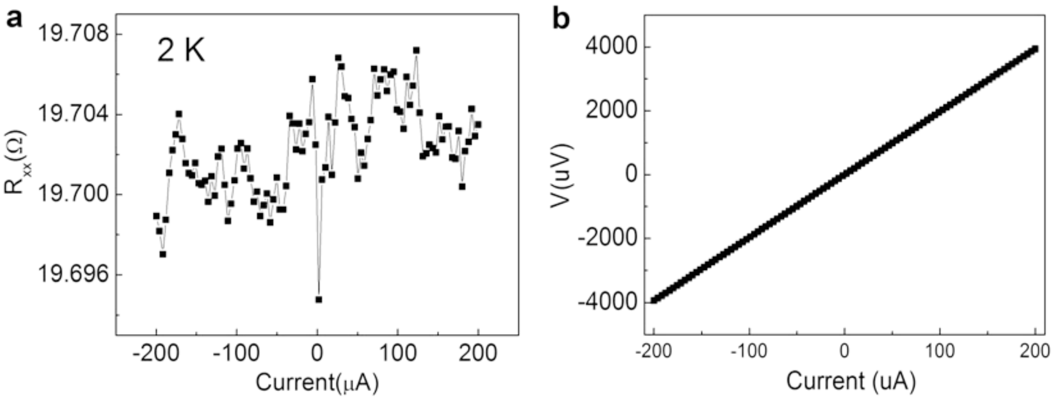

Supplementary Figure 11. (a) R_{xx} vs Current curve at 2 K for an FGT sample. **(b)** Corresponding I-V curve derived from (a).

Supplementary References

1. Gong, C. *et al.* Discovery of intrinsic ferromagnetism in two-dimensional van der Waals crystals. *Nature* **546**, 265–269 (2017).

2. Brown Jr, W. F. Relaxational behavior of fine magnetic particles. *J. Appl. Phys.* **30**, S130–S132 (1959).

3. Kneller, E. & Luborsky, F. Particle size dependence of coercivity and remanence of single-domain particles. *J. Appl. Phys.* **34**, 656–658 (1963).

4. Skomski, R., Oepen, H.-P. & Kirschner, J. Micromagnetics of ultrathin films with perpendicular magnetic anisotropy. *Phys. Rev. B* **58**, 3223 (1998).

5. Fei, Z. *et al.* Edge conduction in monolayer WTe_2. *Nat. Phys.* **13**, 677–682 (2017).
